# A micro-genesis account of longer-form reinforcement learning in structured and unstructured environments

**DOI:** 10.1038/s41539-021-00098-4

**Published:** 2021-06-23

**Authors:** Benjamin James Dyson, Ahad Asad

**Affiliations:** 1 University of Alberta, Edmonton, AB Canada; 2 University of Sussex, Falmer, UK; 3 Ryerson University, Toronto, ON Canada

**Keywords:** Human behaviour, Operant learning

## Abstract

We explored the possibility that in order for longer-form expressions of reinforcement learning (*win-calmness*, *loss-restlessness*) to manifest across tasks, they must first develop because of micro-transactions within tasks. We found no evidence of *win-calmness* or *loss-restlessness* when wins could not be maximised (*unexploitable* opponents), nor when the threat of win minimisation was presented (*exploiting* opponents), but evidence of *win-calmness* (but not *loss-restlessness*) when wins could be maximised (*exploitable* opponents).

## Introduction

Behaviour is dynamically shaped via the reinforcement learning (operant conditioning) principles of *win-stay* and *lose-shift*^1^. The tendency to repeat actions that produce positive outcomes, and, to change actions that produce negative outcomes are fundamentally important associations. First, these close (i.e., trial *n-1* on trial *n*) outcome-action bonds have a stronger effect on performance than any other higher-order bonds (e.g., trial *n-2* on trial *n*; see^2–4^). Second, relatively simple *win-stay/lose-shift* models can outperform more complex reinforcement learning models in accounting for human performance^5–7^. Third, the ability to explicitly represent outcome-action associations that are close in time and space acknowledges the limitations of our cognitive system^8–10^. Therefore, focusing on the immediate association between current outcome and future action is both robust and plausible, from a human information-processing perspective.

Nevertheless, longer-form reinforcement learning principles have been identified that extend the associative chain between multiple outcomes and actions. Specifically, (ref. ^11^ p. 1102) identified *gain-calmness* as the “decreased choice switching following prior tasks producing gains” and *loss-restlessness* as the “increased tendency to switch choices following prior tasks with losses.” Described in this way, *gain-calmness* and *loss-restlessness* represent longer-term effects between tasks whereby the degree of historic success or failure produces shockwaves that determine future performance, with *win-stay* and *lose-shift* mechanisms remaining central. Here we explore the possibility that in order for *gain-calmness* and *loss-restlessness* to manifest *between* tasks, these effects must be available at the end of the previous task, and as such, must develop because of micro-transactions *within* tasks.

Across ten experiments, we used the game Rock, Paper, Scissors (RPS) to test a micro-genesis account of these longer-form reinforcement-learning principles at both a group and individual level. Simple zero-sum games offer a high degree of experimental control, move beyond the restrictions of one-shot responding by allowing for an investigation of repeated decision-making^12^^,^^13^, and, can often be fun to play^14^. Furthermore, RPS represents an intriguing, non-binary paradigm in terms of its relation with traditionally dominant forces of behavioural modification. Specifically, reward mechanisms tend to shape behaviour to a greater degree than punishment mechanisms^12^^,^^15^, such that *win-stay* selections are more frequent than *lose-shift* within certain simple games (see^16^ in the context of cooperative games, see^17^ in the context of Matching Pennies). However, RPS can yield an over-use of *lose-shift* relative to *win-stay* behaviour^18–21^.

Specifically, we operationalise *calmness* as two *stay* responses (once between trial *n-2* and *n-1*, and, then again between trial *n-1* and *n*) and *restlessness* as two *shift* responses (once between trial *n-2* and *n-1*, and, then again between trial *n-1* and *n*) within a block of trials. Thus, evidence for *gain-calmness* is observed at the micro-level if the proportion of *calmness* (*stay-stay*) is highest when the outcome of trial *n-2* is a *win*. Similarly, evidence for *loss-restlessness* is observed at a micro-level if the proportion of *restlessness* (*switch-switch*) is highest when the outcome of trial *n-2* is a *loss*.

In Experiments 1–4, we created game spaces where a computerised opponent operated in accordance with a variant of mixed-strategy, guaranteeing *unexploitability*. This represented an initially unstructured learning environment in which the average win rate would centre on the baseline value determined by the game (i.e., Rock, Paper, Scissors = 33.3%). Since this baseline value is expected regardless of how the participant behaves, standard law of effect mechanisms (*win-stay*, *lose-shift*;^1^) should not operate. This further echoes the caveat that participants need to experience reliable gains or losses (i.e., different from baseline) for *gain-calmness* and *loss-restlessness* to take effect^11^. Therefore, we should not see any evidence of longer-form *stay* or *shift* behaviour determined by trial *n-2* outcome against *unexploitable* opponents, where win rates cannot reliably deviate from baseline. Importantly, participants never encountered any other form of opponent in these studies. Thus, Experiments 1–4 serve as a benchmark as to the degree to which *calmness* and / or *restlessness* is produced by prior outcome when win rates cannot be maximised.

**Fig. 1:**
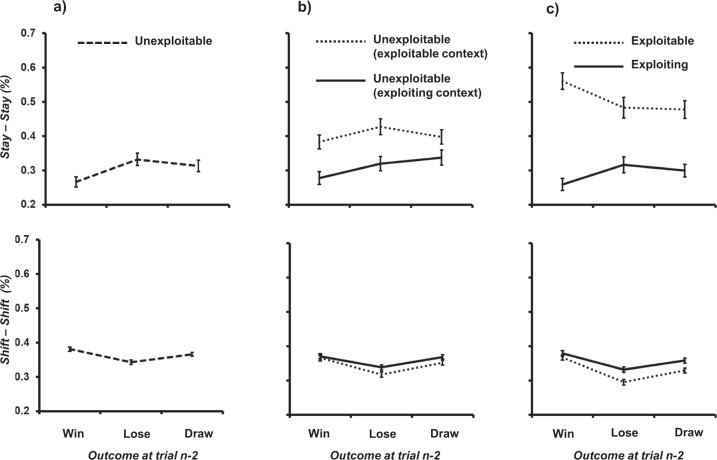
Proportion of calmness (*stay – stay*) and restlessness (*shift – shift*) behaviour between trial *n-2* to *n-1*, and, trial *n-1* to *n* as a function of outcome at trial *n-2* (*win, lose, draw*), and the nature of opponency. Error bars represent standard error. Group performance is represented when participants engaged with **A** *unexploitable* opponents only, **B** *unexploitable* opponents in the context of other opponents, and **C** *exploitable* and *exploiting* opponents.

Participants were rejected from analysis if they failed to exhibit all combinations of trial *n-2* outcome (*win, lose, draw*) x trial *n-1* behaviour (*stay, shift*; missing data prevents the use of a within-participants analysis). Of the initial sample of 148, 145 data sets (97.97%) were retained. In accordance with the *unexploitable* nature of the opponent, the observed win rate across the sample of 33.01% (95% CI [32.39%, 33.62%]) was not significantly different from the expected win rate of 33.33% (*t*[144] = −1.03, *p* = 0.304, *d* = −0.08; one-sample t-test). With respect to the proportion of trials representing *calmness* (here*, x stay – stay*), a one-way repeated-measures ANOVA was conducted where *x* represents *wins*, *losses* and *draws*, a main effect was shown: F(2,288) = 6.76, MSE = 0.024, *p* = 0.001, ƞ_p_^2^ = 0.045. Tukey’s HSD (*p* < 0.05) revealed that *calmness* was significantly *less* likely following wins (26.61%) relative to both losses (33.09%) and draws (30.97%; see Fig. 1A). With respect to the proportion of trials representing *restlessness* (here*, x shift-shift*) where, again, *x* represents *wins*, *losses* and *draws*, a main effect was again shown: F(2,288) = 28.47, MSE = 0.002, *p* < 0.001, ƞ_p_^2^ = 0.165. Tukey’s HSD (*p* < 0.05) revealed that *restlessness* was significantly *less* likely following losses (34.29%) relative to both wins (38.28%) and draws (36.68%). Therefore, neither *win-calmness* nor *loss-restlessness* were apparent under conditions against an *unexploitable* opponent where win rates could not be maximised.

Experiment 5–10 consisted of participants completing 2 blocks against an *unexploitable* opponent (defined as per Experiments 1–4) and either 2 blocks against an *exploiting* opponent (Experiments 5, 7, 9), or, 2 blocks against an *exploitable* opponent (Experiments 6, 8, 10). We operationalized an *exploiting* opponent as one who would take advantage of periodic item biases expressed by the participant, and, an *exploitable* opponent as one who expressed an item bias themselves. This allowed us to answer two further questions. First, we attempt to replicate the absence of *win-calmness* and *loss-restlessness* as a result of *unexploitable* opponency, but within the larger context of other opponent types. Whether the nature of opponency bled into *unexploitable* contexts was deemed a critical consideration, given the putative cross-task expression of *win-stay* and *lose-shift* mechanics^11,14^. Second, we assessed the degree of *win-calmness* and *loss-restlessness* within structured learning environments where win rates could decrease or increase from baseline via engagement with *exploiting* (threat of win minimisation) and *exploitable* (promise of win maximisation) opponency, respectively. If any form of win rate deviation from baseline was sufficient for the expression of *calmness* or *restlessness*, then we should see such behaviour in evidence irrespective of whether the opponent is *exploiting* or *exploitable*. However, if only deflated (inflated) win rate determines *win-calmness* and *loss-restlessness*, then such behaviour should only be observed in the case of *exploiting (exploitable)* opponents.

A total of 208 participants were initially available for analysis from *exploiting* (*n* = 100) and *exploitable* (*n* = 108) experiments. From this initial sample, 84.13% (*n* = 175) were retained for *calmness* and *restless* analysis for *unexploitable* opponents, in accordance with the criteria specified for Experiments 1–4. Observed win rates against *unexploitable* opponents were not significantly difference between the two contexts (32.94% [95% CI [31.43%, 42.85%]] vs. 32.96% [95% CI [29.66%, 39.66%]]): *t*[173] = −0.50, *p* = 0.959, *d* = 0.006 (two-sided, independent t-test). Once again, in accordance with *unexploitable* opponency, neither was significantly different from 33.33% (*t*[81] = −0.98, *p* = 0.328, *d* = −0.107, and, *t*[92] = −1.04, *p* = 0.300, *d* = −0.109; one-sampled t-test).

First, we examined *unexploitable* opponency in the context of other opponents. *Calmness* (here*, x stay – stay*) was once again assessed using the within-participant factor of outcome (*x*: *win*, *lose* and *draw)* and the between-participant factor of context (*exploiting, exploitable*) using a mixed ANOVA. A significant main effect of context: F(1,173) = 12.94, MSE = 0.084, *p* < 0.001, ƞ_p_^2^ = 0.070 revealed increased *calmness* against *unexploitable* opponent during exploitable (40.26%) relative to exploiting (31.14%) contexts. This is clear evidence of *win-calmness* transferring from an *exploitable* opponent to an *unexploitable* opponent^11^. The significant main effect of outcome: F(2,346) = 5.71, MSE = 0.017, *p* = 0.004, ƞ_p_^2^ = 0.032, replicated the observation that calmness was significantly *less* likely following wins (33.35%) relative to both losses(37.69%) and draws (36.91%). There was no significant interaction: F(2,346) = 1.80, MSE = 0.017, *p* = 0.167, ƞ_p_^2^ = 0.010 (see Fig. 1B).

*Restlessness* (here*, x shift - shift*) was analysed in a similar way, revealing only a main effect of outcome: F(2,348) = 35.67, MSE = 0.002, *p* < 0.001, ƞ_p_^2^ = 0.170. Again, *restlessness* was significantly less likely following losses (32.71%) relative to both wins (36.76%) and draws (35.96%; Tukey’s HSD, *p* < 0.05). Neither the main effect of context: F(1,174) = 2.64, MSE = 0.010, *p* = 0.106, ƞ_p_^2^ = 0.015, nor the interaction: F(2,348) = 1.24, MSE = 0.002, *p* = 0.291, ƞ_p_^2^ = 0.007, were significant (see Fig. 1B). Collectively, these data replicate the observations from Experiment 1–4, in that under conditions where win rate minimisation was threatened, neither *win-calmness* nor *loss-restlessness* were apparent.

Second, we directly compared performance between *exploiting* and *exploitable* opponents. 75.48% (*n* = 157) of data were retained for subsequent analysis. Win rates were significantly different between *exploiting* (34.18% [95% CI [33.92%, 46.06%]) and *exploitable* (43.78% [95% CI [58.82%, 81.72%]) opponents: *t*[155] = 10.98, *p* < 0.001, *d* = 1.321 (two-sided, independent t-test). *Exploiting* (*t*[83] = 2.06, *p* = 0.042, *d* = 0.226) and *exploitable* (*t*[72] = 11.65, *p* < 0.001, *d* = 1.527) opponent win rates were different from the expected baseline value of 33.33%. *Calmness* (*stay – stay*) behaviour was examined as a function of the within-participants factor outcome (*win, lose, draw*), and, the between-participants factor opponent (*exploiting, exploitable*) using a mixed ANOVA. The main effect of opponency was significant: F(1,155) = 60.26, MSE = 0.090, *p* < 0.001, ƞ_p_^2^ = 0.280, the main effect of outcome was not significant: F (2,310) = 0.94, MSE = 0.018, p = 0.391, ƞ_p_^2^ = 0.006, and, there was a significant interaction between opponency x outcome: F(2,310) = 11.86, MSE = 0.018, *p* < 0.001, ƞ_p_^2^ = 0.071. *Calmness* was significantly higher against *exploitable* (50.71%) versus *exploiting* (29.16%) opponents. The interaction was caused by *calmness* being numerically less likely following wins (25.88%) relative to losses (31.67%) and draws (29.94%) against *exploiting* opponents (Tukey’s HSD, *p* > 0.05), but significantly more likely following wins (56.05%) relative to both losses (48.30%) and draws (47.78%) against *exploitable* opponents (Tukey’s HSD, *p* < 0.05; see Fig. 1C).

A similar analysis using *restlessness* (*shift - shift*) showed a significant main effect of opponent: F(1,155) = 9.07, MSE = 0.009, *p* = 0.003, ƞ_p_^2^ = 0.055, a significant main effect of outcome: F(2,310) = 58.67, MSE = 0.002, *p* < 0.001, ƞ_p_^2^ = 0.275, but no interaction: F(2,310) = 2.42, MSE = 0.002, *p* = 0.091, ƞ_p_^2^ = 0.015. Here, *restlessness* was greater during *exploiting* opponents (35.62%) relative to *exploitable* opponents (33.00%). As in all other analyses, *restlessness* was also significantly *less* likely following losses (31.44%) relative to both wins (37.32%) and draws (34.43%).

According to^11^, longer-form reinforcement learning associations may arise where tendencies to repeat responding are facilitated by previous exposure to success (*win-calmness*), whereas tendencies to change responding are facilitated by previous exposure to failure (*loss-restlessness*). We tested a micro-genesis account of these longer-form associations by analysing the contingencies between trial triplets (*n-2, n-1, n*) within blocks and as a function of opponency.

We reliably saw that when individuals worked in unstructured learning environments where win rates could not be maximised, neither *win-calmness* nor *loss-restlessness* were in evidence. In fact, the data generated by exclusively *unexploitable* contexts is best characterised as *lose-calmness* and *win-restlessness*. One possibility is that these longer-form reinforcement learning principles are inhibited when standard law-of-effect mechanisms cannot operate (see also *unexploitable* opponent (exploiting context), *exploiting* opponent; Fig. 1), resulting in weak expression of the opposite direction. When *unexploitable* opponents were encountered in the context of other *exploiting* opponents, the latter did not influence behaviour against the former (see Fig. 1B, solid line). However, when *unexploitable* opponents were played in the context of other *exploitable* opponents, the degree of *calmness* rose (see Fig. 1B, dotted line). This shows that the promise of win maximisation—rather than the threat of win minimisation—allows for cognitive transactions across blocks. Fundamental differences between human cognition as a function of win maximisation (rather than win minimisation) was further manifest in the direct comparison between *exploiting* and *exploitable* opponents. Here, *exploiting* opponents yielded data equivalent to that generated by *unexploitable* opponents: *lose-calmness and win-restlessness* (see Fig. 1C, solid line). In stark contrast, when the promise of win maximisation was available via exposure to *exploitable* opponents, *win-calmness* was clearly in evidence at the micro-level (see Fig. 1C, dotted line).

**Fig. 2:**
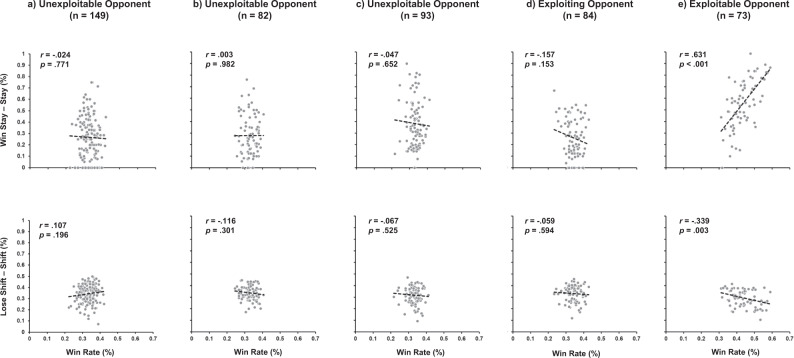
Scatterplots of *win-calmness* (*stay – stay*) and *loss-restlessness* (*shift – shift*) behaviour as a function of individual win rate, and the nature of opponency. Individual performance is represented when participants engaged with **A** *unexploitable* opponents only, **B** *unexploitable* opponents in the context of *exploiting* opponents, **C** *unexploitable* opponents in the context of *exploitable* opponents, **D** exploiting opponents, and **E** exploitable opponents. Only correlations with win rate against *exploitable* opponents were significant *win-calmness* (*r* = 0.631, *p* < 0.001), *loss-restlessness* (*r* = −0.339, *p* = 0.003).

To further support win maximisation as the mechanism by which longer-form reinforcement learning expressions were manifest, we examined performance across all 10 experiments (*n* = 357) at an individual level. Here, the degree of *win-calmness* and *loss-restlessness* was correlated with win rate across the five categories of opponency (*unexploitable*, *unexploitable* in the context of *exploiting*, *unexploitable* in the context of *exploitable*, *exploiting, exploitable*; see Fig. 2A–E). Only against *exploitable* opponents do we observe a positive correlation between *win-calmness* and win rate (*r* = 0.631, *p* < 0.001), and, a negative correlation between *loss-restlessness* and win rate (*r* = −0.339, *p* = 0.003). Moreover, the difference between these correlations was also significantly different (*z* = 6.48, *p* < 0.001).

In sum, the expression of longer-form behavioural *calmness* and *restlessness* is the result of micro-transactions between trials in accordance with classic reinforcement learning principles of *win-stay* and *lose-shift*. That *calmness* and *restlessness* manifest under different opponent contexts (see Fig. 1) and are differentially impacted by win rate (see Fig. 2) at both group and individual levels, is consistent with the multiple anatomical^22^, evolutionary^10,23^ and behavioural^2^ observations that *win-stay* and *lose-shift* are independent.

Specifically, periods of behavioural inertia because of success (*win-calmness*) are much more apparent (and more closely tied to individual win rate experience) than periods of behavioural reconfiguration following failure (*loss-restlessness*). That it should be so. The organism may enjoy the repetition of their opponent’s exploitation during success in structured learning environments that afford success, but must engage in explorative behaviour when the mental model of opponent exploitation is elusive^24^ or they themselves run the risk of exploitation.

Further research will require identifying which characteristics of the decision-making space allow for (a) violations of *win-calmness* and *loss-restlessness* during unsuccessful win maximisation, and (b) provide evidence for *win-calmness* (but not *loss-restlessness*) during successful win maximisation. For example^25^, emphasizes the importance of the kind of structural information participants are provided prior to the game. In contrast^16^, note that patterns of data regarding *win-stay* / *lose-shift* dominance within a cooperative game are largely independent of group size. Similarly, we might speculate whether there is something unique about binary (i.e., 2 response) versus non-binary (i.e., 3+ response) decision-making paradigms. In particular, we need to consider whether *win-stay* preponderance is more likely within binary response games whereas *lose-shift* is more likely within non-binary response games, and, the degree to which restricted choice maps onto aspects of real-world decision-making^13^. Our forthcoming work related to the comparison of structurally isomorphic 3 vs. 5, and, 2 vs. 6 response games should in part help to illuminate game characteristics that play a fundamental role in determining both short- and long-form reinforcement learning principles.

## Methods

### Experiments 1–4

Data from 148 participants were reanalysed from (^18^ Experiment 1) (^19^ Experiment 1), (^20^ Experiment 1) and^21^. See Table 1 for demographic information and block x trial structure. All studies were approved by the Life Sciences and Psychology Research Ethics Committee (C-REC) at the University of Sussex (ER/BJD21/3, ER/BJD21/4), or, Research Ethics Board 2 at the University of Alberta (PRO00083768, PRO00086116). Participants from refs. ^19,21^ provided informed consent from the University of Sussex community, and received either course credit or £20 for participation. Performance-independent compensation was offered as behavioural data were collected in the context of a longer electroencephalographic study. Participants from refs. ^18,20^ were recruited from the undergraduate community at the University of Alberta as part of the Psychology Research Participation scheme, and received performance-independent course credit.

**Table 1:** Summary statistics from those individuals who volunteered demographic information across experiments 1–10. Standard deviation in brackets.

	*n*	Blocks x Trials	Demographic *n*	Female	Age
Experiments 1–4					
Dahal et al. (in review, Experiment 1)	40	1 × 120	37	28	19.03 (1.11)
Dyson et al. (2020; Experiment 1)	36	1 × 225	36	29	21.31 (3.51)
Dyson (2021, Experiment 1)	36	3 × 120	36	25	20.11 (3.27)
Forder & Dyson (2016)	36	3 × 150	36	30	21.22 (3.96)
Experiments 5–10					
Experiment 5	36	4 × 90	35	25	19.06 (2.62)
Experiment 6	36	4 × 90	30	14	18.80 (1.54)
Experiment 7	28	4 × 90	27	16	19.11 (1.89)
Experiment 8	36	4 × 90	36	21	19.25 (1.63)
Experiment 9	36	4 × 90	35	24	18.89 (1.64)
Experiment 10	36	4 × 90	36	25	19.28 (1.45)

The game Rock, Paper, Scissors represents a competitive, two-player game where each player must select one of three possible responses. When played physically, these responses are variations in hand shape: Rock (closed fist), Paper (flat hand), Scissors (index and middle fingers extended and separated). In the current computerised version of the game, participants selected a key that represented one of these three responses. The rules of the game dictate that if both players select the same response, then the round results in a draw. However, players can win or lose following the additional rules that Rock beats Scissors, Scissors beats Paper, and, Paper beats Rock. Thus, if the responses differ between players, one individual will win and one individual will lose.

In all cases, participants played Rock, Paper, Scissors against an *unexploitable* opponent where wins, losses and draws were assigned the numeric value +1, −1 and 0, respectively. Unexploitability was operationalized by distributing Rock, Paper, Scissors responses equally across the block size (33.3%) but in random order. Pictures of two gloved hands representing the 9 interactions between participant and opponent during Rock, Paper, Scissors were used (approximate on-screen size 10.5 cm x 4 cm). Stimulus presentation and response monitoring was conducted by Presentation software.

### Experiment 5–10

Experiment 5–10 consisted of participants completing 2 blocks against an *unexploitable* opponent (defined as per Experiments 1–4) and either 2 blocks against an *exploiting* opponent (Experiments 5, 7, 9), or, 2 blocks against an *exploitable* opponent (Experiments 6, 8, 10). Conditions were presented across participants in a counterbalanced order, dictated by a Latin square design. In both *exploiting* blocks, the computer would generate a response matrix inverse to the participant’s response selections every 3 trials (after^20^). For example, if the participants played Rock on the first 3 trials, the computer would play Paper on the next 3 trials and so on. In both *exploitable* blocks, the computer expressed the same bias for one of the items (66.67%), with the specific item bias also counterbalanced across participants. Additional manipulations involving display information in Experiments 5–10 were collapsed for the purposes of this analysis. All studies were approved by the Research Ethics Board 2 at the University of Alberta (PRO00087988). Participants provided informed consent from the undergraduate community at the University of Alberta as part of the Psychology Research Participation scheme, and received performance-independent course credit.

Following response selection, RPS selections were displayed for opponent (on the left; blue glove) and / or participant (on the right; white glove) for 1000 ms. This display was removed for 500 ms and then the outcome of the trial was presented for 1000 ms in the form of ‘WIN’ (+1; green font), ‘LOSS’ (−1; red font), or ‘DRAW’ (0; yellow font) as appropriate. The outcome was removed and the player’s score was updated across a 500 ms period, after which the next trial began with a response selection prompt.

### Reporting summary

Further information on research design is available in the Nature Research Reporting Summary linked to this article.

## Supplementary information

Reporting Summary

Supplementary Data 1

## Data Availability

The authors declare that the data supporting the findings of this study are available within the supplementary information files.
