# Supplementary material for: A micro-genesis account of longer-form reinforcement learning in structured and unstructured environments
Source: NPJ Sci Learn. 2021 Jun 23;6:19. doi: 10.1038/s41539-021-00098-4 (PMC8222288; doi:10.1038/s41539-021-00098-4)
Supplement: Supplementary file 1 — Reporting Summary [file 41539_2021_98_MOESM1_ESM.pdf]

nature research

Corresponding author(s): Ben Dyson
Last updated by author(s): May 21, 2021

Reporting Summary

Nature Research wishes to improve the reproducibility of the work that we publish. This form provides structure for consistency and transparency in reporting. For further information on Nature Research policies, see our Editorial Policies and the Editorial Policy Checklist.

Statistics

For all statistical analyses, confirm that the following items are present in the figure legend, table legend, main text, or Methods section.

n/a	Confirmed	
☐	☒	The exact sample size (*n*) for each experimental group/condition, given as a discrete number and unit of measurement
☐	☒	A statement on whether measurements were taken from distinct samples or whether the same sample was measured repeatedly
☐	☒	The statistical test(s) used AND whether they are one- or two-sided<br>*Only common tests should be described solely by name; describe more complex techniques in the Methods section.*
☒	☐	A description of all covariates tested
☒	☐	A description of any assumptions or corrections, such as tests of normality and adjustment for multiple comparisons
☐	☒	A full description of the statistical parameters including central tendency (e.g. means) or other basic estimates (e.g. regression coefficient) AND variation (e.g. standard deviation) or associated estimates of uncertainty (e.g. confidence intervals)
☐	☒	For null hypothesis testing, the test statistic (e.g. *F*, *t*, *r*) with confidence intervals, effect sizes, degrees of freedom and *P* value noted<br>*Give P values as exact values whenever suitable.*
☒	☐	For Bayesian analysis, information on the choice of priors and Markov chain Monte Carlo settings
☒	☐	For hierarchical and complex designs, identification of the appropriate level for tests and full reporting of outcomes
☐	☒	Estimates of effect sizes (e.g. Cohen's *d*, Pearson's *r*), indicating how they were calculated

Our web collection on statistics for biologists contains articles on many of the points above.

Software and code

Policy information about availability of computer code

Data collection	Data was collected using Presentation (NeuroBS) software
Data analysis	TIBCO Statistica was used to analyze the data

For manuscripts utilizing custom algorithms or software that are central to the research but not yet described in published literature, software must be made available to editors and reviewers. We strongly encourage code deposition in a community repository (e.g. GitHub). See the Nature Research guidelines for submitting code & software for further information.

Data

Policy information about availability of data

All manuscripts must include a data availability statement. This statement should provide the following information, where applicable:
- Accession codes, unique identifiers, or web links for publicly available datasets
- A list of figures that have associated raw data
- A description of any restrictions on data availability

Provide your data availability statement here.

Field-specific reporting

Please select the one below that is the best fit for your research. If you are not sure, read the appropriate sections before making your selection.

☐ Life sciences ☒ Behavioural & social sciences ☐ Ecological, evolutionary & environmental sciences

For a reference copy of the document with all sections, see nature.com/documents/nr-reporting-summary-flat.pdf

Behavioural & social sciences study design

All studies must disclose on these points even when the disclosure is negative.

Study description	Quantitative experimental
Research sample	Participants were recruited from the University of Sussex, (UK) community, and, the undergraduate community at the University of Alberta as part of the Psychology Research Participation scheme.
Sampling strategy	Convenience sample was used. Sample sizes were based on previously published work (approximately 36 per experiment), deemed sufficient to reproduce significant effects shown previously in the lab.
Data collection	Pen and paper were used to record informed consent. Desktop PCs using Presentation software were used to collect data. Participants were either run individually or in small (4) groups, using independent PCs. Only the experimenter was present with the participant(s). Participants were blind to the purpose of the study, experimenters were not aware of the specific hypotheses of win-calmness and loss-restlessness at the time of data collection.
Timing	2015 - 2019
Data exclusions	Participants were rejected from contributing to the data due to data recording errors, counterbalancing errors, suspected inattention [Dyson, 2021] or, if the participants decided not to complete the study. Participants were rejected from analysis if they failed to exhibit all combinations of trial n-2 outcome (win, lose, draw) x trial n-1 behaviour (stay, shift; missing data prevents the use of a within-participants analysis).
Non-participation	No participants declined participation
Randomization	All individual experiments were within-participants. Order of conditions was counterbalanced across participants, order of trials was randomized within blocks.

Reporting for specific materials, systems and methods

We require information from authors about some types of materials, experimental systems and methods used in many studies. Here, indicate whether each material, system or method listed is relevant to your study. If you are not sure if a list item applies to your research, read the appropriate section before selecting a response.

Materials & experimental systems

n/a	Involved in the study	
☒	☐	Antibodies
☒	☐	Eukaryotic cell lines
☒	☐	Palaeontology and archaeology
☒	☐	Animals and other organisms
☐	☒	Human research participants
☒	☐	Clinical data
☒	☐	Dual use research of concern

Methods

n/a	Involved in the study	
☒	☐	ChIP-seq
☒	☐	Flow cytometry
☒	☐	MRI-based neuroimaging

Human research participants

Policy information about studies involving human research participants

Population characteristics	See above
Recruitment	(Experiments 1-4) Participants from (14) and (16) were recruited from the University of Sussex community, and received either course credit or £20 for participation.<br><br>(Experiments 5-10) Participants were recruited from the undergraduate community at the University of Alberta as part of the Psychology Research Participation scheme, and received performance-independent course credit

Ethics oversight	All studies were approved by the Life Sciences and Psychology Research Ethics Committee (C-REC) at the University of Sussex (ER/BJD21/3, ER/BJD21/4), or, Research Ethics Board 2 at the University of Alberta (PRO00083768, PRO00086116, PRO00087988).

Note that full information on the approval of the study protocol must also be provided in the manuscript.
